# Do Not Resonate with Actions: Sentence Polarity Modulates Cortico-Spinal Excitability during Action-Related Sentence Reading

**DOI:** 10.1371/journal.pone.0016855

**Published:** 2011-02-11

**Authors:** Marco Tullio Liuzza, Matteo Candidi, Salvatore Maria Aglioti

**Affiliations:** 1 Department of Psychology, University of Rome “La Sapienza”, Rome, Italy; 2 IRCCS, Fondazione Santa Lucia, Rome, Italy; Royal Holloway, University of London, United Kingdom

## Abstract

**Background:**

Theories of embodied language suggest that the motor system is differentially called into action when processing motor-related versus abstract content words or sentences. It has been recently shown that processing negative polarity action-related sentences modulates neural activity of premotor and motor cortices.

**Methods and Findings:**

We sought to determine whether reading negative polarity sentences brought about differential modulation of cortico-spinal motor excitability depending on processing hand-action related or abstract sentences. Facilitatory paired-pulses Transcranial Magnetic Stimulation (pp-TMS) was applied to the primary motor representation of the right-hand and the recorded amplitude of induced motor-evoked potentials (MEP) was used to index M1 activity during passive reading of either hand-action related or abstract content sentences presented in both negative and affirmative polarity. Results showed that the cortico-spinal excitability was affected by sentence polarity only in the hand-action related condition. Indeed, in keeping with previous TMS studies, reading positive polarity, hand action-related sentences suppressed cortico-spinal reactivity. This effect was absent when reading hand action-related negative polarity sentences. Moreover, no modulation of cortico-spinal reactivity was associated with either negative or positive polarity abstract sentences.

**Conclusions:**

Our results indicate that grammatical cues prompting motor negation reduce the cortico-spinal suppression associated with affirmative action sentences reading and thus suggest that motor simulative processes underlying the embodiment may involve even syntactic features of language.

## Introduction

According to standard cognitive theories, language is processed amodally [1], [2] and in higher-order anatomo-functional systems largely unrelated to sensory and motor networks [3]. However, growing behavioral [4]–[7], neuroimaging [8]–[10], neurophysiological [11]–[15] and neuropsychological [16]–[20] evidence indicates that sensorimotor simulation is at play during a variety of language related tasks. Such evidence brought experimental support to the Embodied Cognition framework [21] according to which action-related concepts are represented within the same brain circuitry responsible for executing the actions linked to the expressed concepts. Beside action-related concepts, also language may be embodied [22], [23], [24], and common representational formats may underpin linguistic and sensorimotor processes [25], [26]. Strong support to the experiential-simulative account of language processing comes from studies on action simulation and posits that the automatic and rapid [27] reactivation of the sensorimotor copy of an action is crucial to enable one to understand its linguistic meaning [22]. Coherently, deficits in reactivating the sensorimotor copy of an action should bring about, for example, impaired performance in semantic tasks. Although the robustness of the classical dissociation between apraxic and aphasic deficits (e.g. [28]–[30]) is not at stake here, many studies have shown that such a pattern has indeed been observed in a variety of patients showing sensorimotor deficits associated to Parkinson disease [31], [32], [20], cortico-basal degeneration [18], subcortico-frontal diseases [33], left frontal atrophy [34] and motor neurone disease [16], [17].

Many of the studies on embodied language processing have focused thus far on the semantics of single words (nouns or verbs) (for instance [12], [35]–[37]). Language comprehension, however, is inherently linked to processing whole sentences that are typically made of different semantic units (at least two, noun and verb) and are organized according to specific syntactic rules. Only recently, have researchers begun to investigate the link between motor knowledge and sentence processing based on grammatical cues [7], [38]–[41].

Relevant to this issue is the case of sentential negation, the basic syntactic feature that reverses the truth value expressed by a sentence. Two recent fMRI studies have explored the effect of action negation on brain activity [39], [41]. Tettamanti and colleagues [39] showed that passively listening to negative action related sentences brought about a selective decrease of the BOLD signal in the same fronto-parietal network that was activated by the affirmative form of the same sentences. This decrease was not observed when participants were engaged in abstract sentences listening. In a similar vein, the visual presentation of hand action-related verbs induced higher neural activity in the motor and premotor cortices when the stimuli were positive rather than negative imperatives [41].

S-p TMS studies showed that imagination [42], [43] or direct observation of actual [44]–[48] or implied [49]–[51] actions induced an increase of MEPs amplitude. Such a facilitation effect was highly specific for the muscles that would be involved in actual execution of the observed action [43]–[47], [50], [15] and was likely due to the activity of the fronto-parietal mirror system [48], [52], [53]. This may seem at odds with neurophysiological and behavioral results showing that listening to limb action verbs (e.g., grasp or kick) inhibits the corticomotor representation of the limb involved in the execution of the represented action [14]. However, while the former condition typically provides explicit cues about the properties of a specific action (e.g., movement direction or the specific muscle involved in the action), verbs may typically involve a number of different ways of performing a given action. Therefore, while the facilitation during direct observation may derive from a resonant mirror mapping between model and onlooker, the inhibition during higher-order linguistic derivation may arise from the competition between different motor schemata associated with what is heard or read [14]. In the present study we tested, for the first time using TMS, whether reading sentences that negate or affirm the execution of an action would differentially influence the excitability of the cortico-spinal system.

Using Transcranial Magnetic Stimulation (TMS) we tested the effect of sentential negation on the reactivity of the motor system by assessing any selective modulation of the cortico-spinal excitability during reading affirmative and negative hand action-related sentences. We recorded the amplitude of Motor Evoked Potentials (MEPs) from a hand muscle (First Dorsal Interosseus, FDI) of healthy participants who silently read affirmative and negative polarity, hand action-related and abstract sentences. Furthermore, to functionally characterize any neurophysiological effect contingent upon linguistic negation, we used paired-pulse (pp-) instead than single pulse (sp-) TMS. It is worth noting that, while the effect of sp-TMS may take place at both the motor cortex and the spinal cord level [54], pp-TMS provides a reliable index of selective motor cortical activation. Indeed the MEP facilitation to pp-TMS likely occur at the cortical level and reflects the activation of excitatory cortical interneurons without affecting spinal circuits [55]. Moreover, we chose to use the pp-TMS procedure also on the basis of a previous study showing that pp-TMS (and not sp-TMS) was able to detect modulations of the cortico-spinal system contingent upon processing of hand-action related nouns and verbs [35]. The task was based on the visual presentation of written sentences in order to test the cortico-spinal excitability while subjects were reading the whole sentence (i.e. compositional mechanisms of language) instead of hearing the verb, as in [14].

## Materials and Methods

### Ethics statement

The experimental procedures were approved by the Fondazione Santa Lucia Ethics Committee (24/11/2008) and were carried out in accordance with the principles of the 1964 Declaration of Helsinki.

### Participants

Fourteen individuals (8 males) participated in the study (mean age 23±2.5 SD). All participants were Italian native speaker, were right-handed according to the Standard Handedness Inventory [56] and had normal or corrected-to-normal visual acuity. All participants gave their written informed consent prior to their inclusion in the study and were naive as to its purpose. Participants were compensated for their time, and specific information concerning the study was provided to them only after they had finished all experimental sessions. None of the participants had a history of neurological, psychiatric, or other medical problems or any contraindication to TMS [57]. No discomfort or adverse effects during pp-TMS were noticed or reported.

### Stimuli

During the experimental sessions participants were presented with Italian four-words sentences (see Table 1 for a complete list of stimuli). The sentences were chosen from a set of 60 sentences used in a previous study [39] and adapted to the purpose of the study. The sentences could refer to either abstract activities or hand-action related actions (“Io sogno la pace” which translated in English reads as “I dream the peace”, “Io colgo la mela” which translated in English reads as “I grasp the apple”). Each sentence was presented in both affirmative or negative polarity (“Io spremo il limone” which translated in English reads as “I squeeze the lemon” and “Non spremo il limone” which translated in English reads as “I don't squeeze the lemon”). It is important to note that, in Italian, the negative version of these sentences implicitly includes reference to the first person and thus affirmative and negative sentences are matched for length and reference to the agent of the action. To further control for any possible difference between motor hand-related and abstract items that could affect sentence reading speed we controlled that the frequency of the verb, frequency of the object complement, number of the syllables of the verb, number of syllables of the sentence were accurately matched between categories (according to the corpus provided by the CoLFIS (Corpus e Lessico di Frequenza dell'Italiano Scritto) elaborated by the Computational Linguistics Insititute, National Centre of Research (CNR) and available at http://www.ge.ilc.cnr.it/lessico.php on a database of 3.798.275 words). Conversely to control that items of the two categories differed for their imageability and motor relatedness we asked an independent group of 20 individuals (mean age 27.15±3.87 SD) to rate each experimental item, by marking a 1 to 7 Likert scale, for 1) how fast is the sentence in evoking a mental image, a visual representation, a sound or other perceptual experiences, and 2) how much movement is implied by each sentence (all mean values of these measures are reported in Table 2 and Table S1 of the Supporting Information). Crucially for the purposes of our experiment, action related sentences were more imaginable (6.06±0.89 SD vs 2.44±1.06 SD; *t*(19) = 18.40, *p*<0.001) and more motor related (5.74±1.14 SD vs 1.08±0.15 SD; *t*(19) = 18.04, *p*<0.001) than abstract ones.

**Table 1:** List of all experimental stimuli.

Abstract		Hand action-related	
Positive	Negative	Positive	Negative
Io invidio la bellezza	Non invidio la bellezza	Io afferro la maniglia	Non afferro la maniglia
*I envy beauty*	*I don't envy beauty*	*I grab the handle*	*I don't grab the handle*
Io sogno la pace	Non sogno la pace	Io spremo il limone	Non spremo il limone
*I dream the peace*	*I don't dream the peace*	*I squeeze the lemon*	*I don't squeeze the lemon*
Io rispetto il patto	Non rispetto il patto	Io avvito il bullone	Non avvito il bullone
*I respect the deal*	*I don't respect the deal*	*I screw in the bolt*	*I don't screw in the bolt*
Io tollero lo sgarbo	Non tollero lo sgarbo	Io impugno la spada	Non impugno la spada
*I tolerate the rudeness*	*I don't tolerate the rudeness*	*I clasp the sword*	*I don't clasp the sword*
Io perdono la colpa	Non perdono la colpa	Io colgo la mela	Non colgo la mela
*I forgive the guilt*	*I don't forgive the guilt*	*I pick the apple*	*I don't pick the apple*
Io ricordo il passato	Non ricordo il passato	Io ritaglio la foto	Io ritaglio la foto
*I remember the past*	*I don't remember the past*	*I cut out the picture*	*I cut out the picture*

In *italic* the English translation of each sentence used as stimulus.

**Table 2:** List of frequency, length and subjective ratings' mean values of all stimuli.

	V F	O C F	V No S	S No S	IMAG	MOT R
**Hand Action-related sentences**	70.5±73.7	99.8±127.8	2.7±0.5	7.2±0.8	**6.1±0.9**	**5.7±1.1**
**Abstract content sentences**	353.5±517.7	294.3±212.0	2.8±0.4	7.2±0.8	**2.4±1.1**	**1.1±0.2**
***T value***	*1.33*	*1.92*	*0.62*	*0*	18.4*	18.03*
***P value***	*0.21*	*0.08*	*0.55*	*1*	***<0.001***	***<0.001***

Values represent mean ± standard deviations of stimuli of all experimental conditions.

In *italic* T-tests and *p* values. In bold means that differ significantly between abstract and hand-related sentences**. V F**  =  Verb Frequency; **O C F**  =  Object Complement Frequency; **V No S**  =  Verb Number of Syllables; **S No S**  =  Sentence Number of Syllables; **IMAG**  =  Imageability; **MOT R**  =  Motor Relatedness.

#### Electromiographic Recordings and Transcranial magnetic stimulation

Electromyography (EMG) - MEPs to pp-TMS of the left motor cortex were recorded from the right FDI. Silver/silver chloride surface electrodes were placed over the muscle belly (active electrode) and over the associated joint or tendon of the muscle (reference electrode). A ground electrode was placed on the right wrist. A CED Power 1401 (Cambridge Electronic Design Ltd, Cambridge, UK) was connected to an Isolated Amplifier System Model D360 (Digitimer Limited, Hertfordshire, UK) and interfaced with CED Spike 2 software. The second-order Butterworth filter was set between 20 and 2.5 kHz (sampling rate, 10 kHz). Signals were displayed at a gain of 1000. Auditory feedback of the electromyography signal was used to help subjects maintain voluntary muscle relaxation during electrophysiological preparation.

#### Transcranial Magnetic Stimulation (pp-TMS)

The optimal scalp position (OSP) for inducing MEPs in the right FDI muscle was found by moving the coil in steps of 1 cm over the left primary cortex until the largest MEPs were found. Then, the position was marked with a pen on a bathing cap worn by participants. The coil was held tangential to the scalp with the handle pointing backward and laterally at 45° from the midline. Resting motor threshold (rMT) was defined as the lowest stimulus intensity that evoked at least 5 MEPs out of 10 consecutive magnetic pulses with an amplitude >50 µV. During the experimental blocks, two pulses of TMS were delivered over the individual OSP by connecting two Magstim Model 200 stimulators with a Bistim module (The Magstim Company), producing a maximum output of 1.75 T at the coil surface (stimulus attenuation, 22%; duration, 1 ms; rise time, 110 µs). The two pulses were delivered by means of a 70 mm figure eight stimulation coil (Magstim polyhurethane-coated coil). In standard pp-TMS protocols, a conditioning stimulus (CS) below the rMT, is followed at short interstimulus intervals (ISIs) by a suprathreshold test stimulus (TS). At ISIs of 7–20 msec the CS produce an MEP facilitation which is thought to take place at the cortical level reflecting the activation of excitatory cortical interneurons without affecting spinal circuits [55]. In our study, the CS stimulus was set to 80% of the rMT while the TS pulse was set at 120% of rMT. Mean rMT was 50±SD 9% of maximum stimulator output. The time delay between the first conditioning pulse and the test one was set to 10 ms as this interval has been proven to measure the effect of facilitatory interneuron connections [55]. EMG recording started 100 ms before the test magnetic pulse in order to control for the absence of muscular preactivation in each trial. MEPs' peak-to-peak amplitudes (in millivolts) were collected and stored in a computer for off-line analysis.

### Procedure

**Figure 1:**
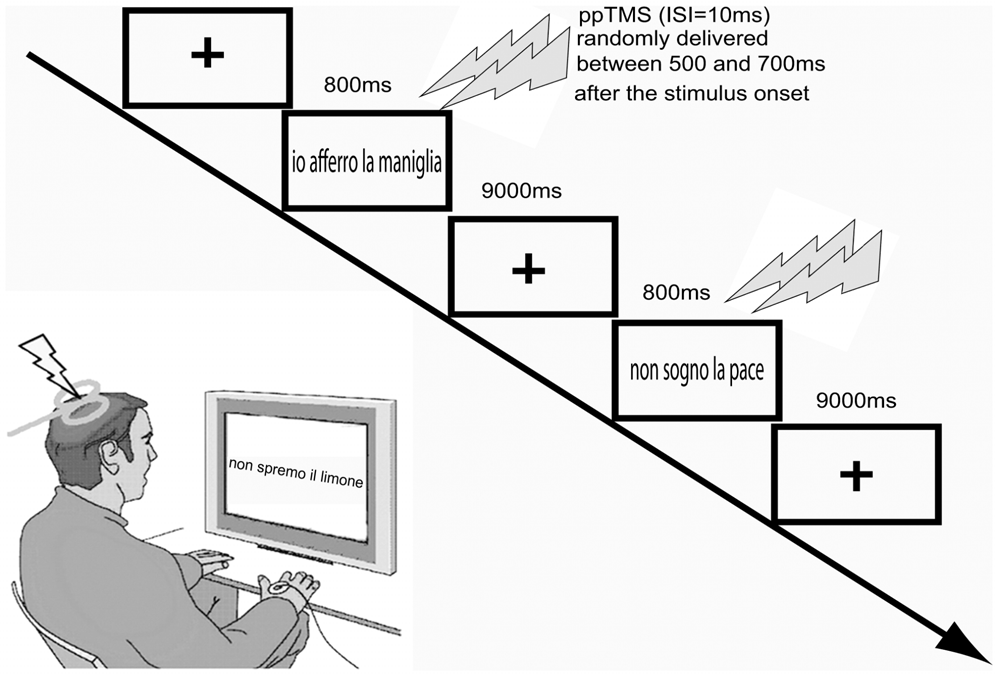
Experimental Procedure. Timeline and subjects' posture during the experimental procedure. Paired-pulse Transcranial Magnetic Stimulation (p-pTMS) was delivered on average 600 ms (±100 ms) after each sentence appeared on the screen. Stimulation intensity was based on individual resting motor threshold (rMT) for the first dorsal interosseous (FDI). The Conditioning Stimulus (CS) was set at an intensity of 80% of rMT while the Test Stimulus (TS) at 120% of rMT with an Inter Stimulus Interval (ISI) of 10 ms over FDI Optimal Scalp Position (OSP).

Participants sat with their right and left arm and hand resting on a pillow on their lap. The participants were comfortably seated in a dimly lit room at a distance of 80 cm from a computer screen. Eighteen abstract and eighteen motor (nine positive and nine negative) sentences were randomly presented within each of the two experimental blocks, intermingled with the presentation of nine black squares which enabled us to measure the baseline cortico-spinal excitability of the hand muscle (45 trial per block for a total of 18 trials per condition). At the beginning of the experiment, subjects were instructed to pay attention to the visual stimuli presented on the screen as, during the inter-trial interval, they would be asked questions concerning the last read sentence (ITI 10 s). For example subject could have been asked whether the sentence was positive, negative, whether the last word ended with an “a” or not and to answer to questions about the meaning of the sentence as “Do you grasp the apple?”, “Do you respect the deal?”. The choice of the duration of this inter-trial interval was based on research [58] that showed no change in cortico-spinal excitability after repetitive TMS at 0.1 Hz for 1 h. This procedure allowed us to rule out that effects of TMS per se influenced the results. Each trial started with a fixation cross lasting 10 s followed by the presentation of the sentence or the fixation square which lasted 800 ms. During the presentation of each sentence (or black square) a paired-pulse TMS was delivered at randomly variable time intervals ranging between 500 and 700 ms after stimulus onset. The decision to stimulate the cortico-spinal system in this time window was based on Pulvermüller's neurophysiological research showing early (200 ms) EEG modulations over central sites during action related-verbs and nouns reading respectively, and later (500–800 ms) high frequency (30 Hz) modulations recorded from central sites (C3/C4) for action verbs compared to nouns [12]. A schematic representation of two different-stimulus category trial events is shown in Figure 1.

### Data Analysis

MEP amplitudes that fell 3 SDs above or below each individual mean for each experimental condition or single trials contaminated by muscular preactivation were excluded as outliers and precontracted trials, respectively (5% of total). Raw MEP amplitudes for each condition were normalized (divided) by baseline MEP amplitudes. Normalized MEP amplitudes were entered in a 2 (sentence Type: Abstract, Hand-related) X 2 (Polarity: Negative, Positive) repeated measures ANOVA. Post-hoc analysis was performed with Duncan test. All statistical tests were performed with the software STATISTICA 8 (StatSoft, Tulsa, OK, USA).

## Results

**Figure 2:**
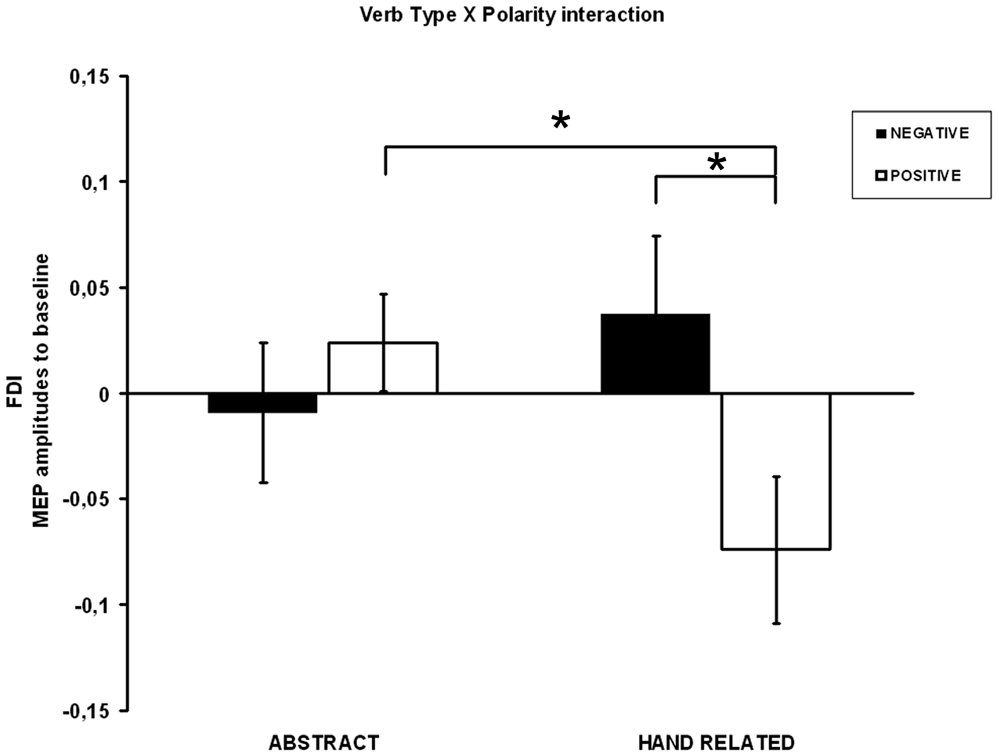
MEP amplitudes in all experimental conditions. MEP amplitudes are represented with respect to their baseline value of excitability recorded during the observation of a black square (normalized MEP-1). Motor potentials evoked during positive hand action related sentences were significantly inhibited with respect to the ones evoked during positive abstract sentences and with respect to baseline. MEPs were modulated by linguistic polarity during hand action related sentences comprehension but not during abstract sentences. Vertical bars denote standard error means.

Analysis of MEP amplitudes revealed a main effect of Polarity (*F*(1,13) = 4.94, *p* = 0.045, η^2^ = 0.27) which was accounted for by smaller MEP amplitudes during reading positive than negative sentences (0.97±SD 0.12 vs 1.01±SD 0.13, note that in Figure 2 the MEP amplitudes are reported with respect to the baseline value, normalized MEP-1). The sentence Type main effect was non significant (*F*(1, 13) = 2.88, *p* = 0.113, η^2^ = 0.18). Importantly, the sentence Type by Polarity interaction was significant (*F*(1,13) = 5.77, *p* = 0.032, η^2^ = 0.31) (see Figure 2).

Post-hoc comparisons revealed that the interaction was entirely accounted for by a suppression of cortico-spinal excitability during hand related positive sentences. Indeed, reading positive hand related sentences induced lower cortico-spinal excitability (0.93±SD 0.13) with respect to both reading positive abstract sentences (1.02±SD 0.09, p = 0.047, Cohen's *d*  = 0.88) and negative hand related sentences (1.04±SD 0.14, p = 0.030, Cohen's *d*  = 0.84). Furthermore, the almost significant trend showed by the T-test against baseline (value 1) (*t*(13) = −2.13, *p* = 0.053) indicated that the MEP suppression during reading positive hand related sentences was a genuine inhibition of the cortico-spinal excitability. All other conditions did not differ from one another (all *ps*>0.15) and did not differ from baseline (*ps*>0.1).

## Discussion

### Cortico-spinal signatures of motor simulation are found when reading action-related but not abstract sentences

The first result of our study is the decrease of MEP amplitudes when subjects silently read positive action-related sentences compared to positive abstract sentences. This is in keeping with a previous sp-TMS study where subjects listened to auditory verbal stimuli and they received a single pulse at the end of the verb [14]. Our pp-TMS results expand previous knowledge by demonstrating that the effect has to do more with to cortical interneurons than with modulations at any other level of the cortico-spinal pathway [54]. Moreover, unlike a previous study using auditory presentation of single words [14], we used visual presentation of sentences that allowed us to test language processing at the sentence level and thus the effects of the compositional mechanisms of language more than the processing of the single verb [14]. This latter aspect of our study is crucial in order to test the theories postulating that sensorimotor simulation contributes to language processing.

### Lack of action simulation during processing of negative polarity, hand action-related sentences

The key result of our TMS study is that sentential polarity selectively modulates cortico-motor reactivity only when the sentence refers to hand actions. Psycholinguistic studies show slower reactivity to stimuli referred to in a negative sentence suggesting a sort of experiential based language comprehension [59]. Studies on the neural basis of negation were performed mainly using functional neuroimaging techniques [60], [61]. An fMRI study focusing on the neural basis of bilingualism, for example, reported that neural activity in parietal and frontal regions was higher when listening to negative action-related sentences with respect to positive ones [61]. However, the study did not use non action-related, control stimuli. Moreover, the effect was present only when processing the participants' second language and it was interpreted as being related to the difficulty of the task and not in terms of motor simulation vs. no-simulation [61]. More recently, two fMRI studies specifically tested the effect of sentential negation in relation to the language-mediated embodiment of actions [39], [41]. Passive listening of action-related or abstract sentences uttered in affirmative or negative polarity demonstrated that processing of negative action-related sentences brought about a reduction of neural activation and cortical connectivity in a left-hemispheric frontoparieto-temporal network [39]. Using a region of interest (ROI) analysis, it has also been shown that visual presentation of negative polarity, imperative action-related verbs induced a reduction of neural activity in motor and premotor regions [41]. Our study complements and expands previous fMRI results for a number of reasons.

By using a pp-TMS procedure we have been able to highlight the specific role played by facilitatory cortico-cortical connections in the action simulation process associated to the representation of grammatical features. More specifically, we found a suppression of MEP amplitudes during positive hand action sentences reading compared with baseline.

Many of the previous findings on the involvement of the motor system in action-related word comprehension have been explained by an associative learning model [11], [37], [62], [63] which posits that action-related verbs automatically co-activate neuronal ensembles dedicated to language and actions. This co-activation would be developed during individuals' ontogenesis as we learn to utter action-related verbs while performing the same actions. However, we show here that such “language-to-motor” neural spread of co-activation is not observed when reading negative forms of action verbs.

Assuming that effective use of cognitive and neural systems is based on the implementation of the exact amount of resources required by the task at hand, it has been postulated that language-mediated motor simulation occurs only when the action is within the linguistic focus [7], [64]. The Linguistic-Focus Hypothesis, in fact, postulates that engagement of the motor system during language comprehension is mediated by the focus of the linguistic message [64]. Taylor and Zwaan [64] used an action compatibility effect experimental paradigm (ACE, [4]) in which participants had to read sentences like “The runner/was very/thirsty./A fan/handed him/a bottle/of cold/water/which he/opened/quickly” in a self paced manner and had to turn a knob in order to proceed in reading the sentence either in a clockwise or in a counter-clockwise direction. The action described in the sentence could either match or mismatch the action that subjects had to perform in order to proceed in reading the sentence. For instance, since opening a bottle of water requires a clockwise action, reading a sentence which describes this action, should induce slower reading times when the knob has to be turned in a counter-clockwise direction because the motor resonance, activated by the verb, interferes with the action to perform in order to execute the task. Importantly, adverbs should modulate the ACE effect only if they deal with the action itself by increasing the linguistic focus on the motor content of the sentence. In fact, their results showed that when a verb was modified by an adverb, compatible motor responses were facilitated when reading the adverb only if the adverb primarily modified an action-related feature (e.g., quickly and slowly) and not when some other element of the referential situation was modified (e.g. happily, eagerly, or nervously). Within this theoretical framework, we propose that sentential negation is a powerful grammatical cue that could suppress the sensorimotor simulation of the (negated) action. The neural counterpart of such mechanism may be the lack of reduction of cortico-motor resonance for negative action verbs.

Psychophysical studies on the effects of the representation of linguistic negation suggest that the temporal characteristics of the experimental task (i.e. fast or delayed decision) have different effects on the processing of ‘what is negated’ [65]. On the basis of this and other behavioral findings [66], Kaup and colleagues [66], [59] have proposed a two-step model of negation processing in which comprehenders first create a representation of ‘what’ is negated and than shift their attention towards the actual state of affairs (the state implied by the negation) at a later point in the comprehension process. On the basis of their data, the first step seems to occur within the first 1500 ms after the sentence onset, the second step should occur after 1500 ms or later. Interestingly, the fine-grained temporal resolution provided by TMS allowed us to provide neural indexes of the lack of simulation contingent upon negation even in the time window where affirmative and negative sentences should not differ on the basis of the model proposed by Kaup and colleagues [59] (500–700 ms after stimulus presentation).

### Conclusions

In conclusion, our results demonstrate a selective modulation of the cortico-spinal excitability during: i) reading positive action related sentences with respect to positive, non-action related sentences; and ii), more importantly, reading positive hand action-related sentences compared with action-related negative sentences. Thus, we show that negation does not play a non-specific role in sentence representation but it does act as a gate that inhibits cortico-spinal sensorimotor simulation.

## Supporting Information

Table S1 **List of all experimental sentences.** Each sentence is reported with its associated value of: **V F**  =  Verb Frequency; **O C F**  =  Object Complement Frequency; **V No S**  =  Verb Number of Syllables; **S No S**  =  Sentence Number of Syllables; **IMAG**  =  Imageability; **MOT R**  =  Motor Relatedness. In *italic* the English translation of each sentence. Frequencies are absolute values in the CoLFIS database (http://www.ge.ilc.cnr.it/lessico.php).
(DOCX)
