# Supplementary material for: Do Not Resonate with Actions: Sentence Polarity Modulates Cortico-Spinal Excitability during Action-Related Sentence Reading
Source: PLoS One. 2011 Feb 11;6(2):e16855. doi: 10.1371/journal.pone.0016855 (PMC3037953; doi:10.1371/journal.pone.0016855)
Supplement: Table S1 — List of all experimental sentences. Each sentence is reported with its associated value of: V F = Verb Frequency; O C F = Object Complement Frequency; V No S = Verb Number of Syllables; S No S = Sentence Number of Syllables; IMAG = Imageability; MOT R = Motor Relatedness. In italic the English translation of each sentence. Frequencies are absolute values in the CoLFIS database (http://www.ge.ilc.cnr.it/lessico.php). (DOCX) [file pone.0016855.s001.docx]

## Supporting Information

**Table S1. List of all experimental sentences.**  Each sentence is reported with its associated value of: **VF** = Verb Frequency; **OCF** = Object Complement Frequency; **VNoS** = Verb Number of Syllables; **SNoS** = Sentence Number of Syllables; **IMAG** = Imageability; **MOT R** = Motor Relatedness. In *italic* the English translation of each sentence. Frequencies are absolute values in the CoLFIS database (<http://www.ge.ilc.cnr.it/lessico.php>).

| **Hand Action-related sentences** | **VF** | **OCF** | **VNoS** | **SNoS** | **IMAG** | **MOT R** |
| --- | --- | --- | --- | --- | --- | --- |
| Io afferro la maniglia/ *I grab the handle* | 126 | 15 | 3 | 8 | 6.58 | 5.63 |
| Io avvito il bullone/*I screw the bolt* | 6 | 9 | 3 | 8 | 5.8 | 5.85 |
| Io colgo la mela/*I pick the apple* | 194 | 66 | 2 | 6 | 5.8 | 5.8 |
| Io impugno la spada/*I clasp the sword* | 46 | 55 | 3 | 7 | 5.4 | 4.5 |
| Io ritaglio la foto/*I cut the picture* | 27 | 351 | 3 | 7 | 6.2 | 6.05 |
| Io spremo il limone/*I sqeeze the lemon* | 24 | 103 | 2 | 7 | 6.63 | 6.5 |
| **Abstract content sentences** | **V F** | **O C F** | **V No S** | **S No S** | **IMAG** | **MOT R** |
| Io invidio la bellezza/ *I envy beauty* | 46 | 294 | 3 | 8 | 1.9 | 1.1 |
| Io perdono la colpa/ *I forgive the guilt* | 111 | 312 | 3 | 7 | 2 | 1.05 |
| Io ricordo il passato/ *I remember the past* | 1392 | 617 | 2 | 8 | 3.8 | 1.2 |
| Io rispetto il patto/ *I respect the deal* | 268 | 139 | 3 | 7 | 2.2 | 1.1 |
| Io sogno la pace/ *I dream the peace* | 251 | 401 | 2 | 6 | 2.5 | 1 |
| Io tollero lo sgarbo/ *I tolerate the rudeness* | 53 | 3 | 3 | 7 | 2.25 | 1.05 |
